# Porto-mesenteric four-dimensional flow MRI: a novel non-invasive technique for assessment of gastro-oesophageal varices

**DOI:** 10.1186/s13244-024-01805-6

**Published:** 2024-09-27

**Authors:** Rasha Karam, Basma A. Elged, Omar Elmetwally, Shahira El-Etreby, Mostafa Elmansy, Mohammed Elhawary

**Affiliations:** 1 Department of Radiology, Mansoura University, Mansoura, Egypt; 2 Department of Internal Medicine, Hepatology and Gastroenterology Unit, Mansoura University, Mansoura, Egypt

**Keywords:** Portal hypertension, Varices, 4D flow MRI, Risk stratification

## Abstract

**Objectives:**

To assess the role of 4D flow MRI in the assessment of gastro-oesophageal varices and in the prediction of high-risk varices in patients with chronic liver disease.

**Methods:**

Thirty-eight patients diagnosed with either oesophageal or gastric varices were included in this single-centre prospective study. 4D flow MRI was used to calculate peak flow, average flow and peak velocity at the portal vein confluence (PV1) and hilum (PV2), splenic vein hilum (SV1) and confluence (SV2), and superior mesenteric vein (SMV). PV and SV fractional flow changes were also measured.

**Results:**

ROC analysis revealed that both PV2 average flow and PV fractional average flow change had 100% sensitivity to predict high-risk patients with the PV fractional peak flow change having the widest area under the curve (AUC) and the highest specificity (92.3%). SV1 average flow, SV2 average flow, SV2 peak flow, and SV2 peak velocity increased significantly in patients with oesophageal compared to gastric varices included (*p* = 0.022, < 0.001, < 0.001 and 0.001, respectively).

**Conclusion:**

Based on certain porto-mesenteric blood flow, velocity, and fractional flow change parameters, 4D flow MRI showed excellent performance in identifying high-risk patients and giving an idea about the grade and location of varices.

**Critical relevance statement:**

Variceal bleeding is a major consequence of unidentified risky upper GI varices. Thus, by identifying and locating high-risk varices early, either oesophageal or gastric, using a non-invasive method like MRI, adverse events might be avoided.

**Key Points:**

- 4D flow MRI can be used as a potential alternative for endoscopy to predict patients with high-risk varices.
- Based on portal vein fractional flow change, splenic flow and velocity, 4D MRI can predict and locate high-risk varices.
- Earlier identification of high-risk varices can allow for interventions to prevent adverse events.

**Graphical Abstract:**

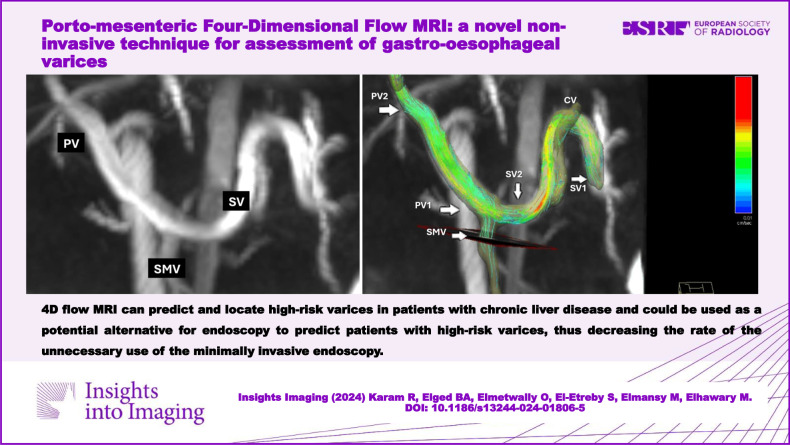

## Introduction

Liver cirrhosis is a major health problem in Egypt, as it contributes to 103.3 per 100,000 deaths, making Egypt the country with the highest cirrhosis-related mortality [1]. The main aetiology of that condition in Egypt is viral hepatitis, especially the hepatitis C virus (HCV), which has a pooled prevalence of 11.9% in the Egyptian population [2]. Liver cirrhosis results in increased intrahepatic vascular resistance, causing increased venous pressure in the porto-mesenteric vasculature. That condition is defined as portal hypertension, and it is responsible for dreadful cirrhosis-related complications like ascites, hepatic encephalopathy, and variceal bleeding [3, 4].

Variceal bleeding is the most dreadful complication of portal hypertension [5, 6]. Currently, esophagogastroduodenoscopy is the best modality used for diagnosis, risk stratification, and management of upper gastrointestinal (GI) varices [7, 8]. Although it is a minimally invasive method, it requires patient sedation and carries a risk of potential complications like perforation, bleeding, or infection. Therefore, it is crucial to seek a non-invasive alternative modality for risk stratification of oesophageal varices to avoid adverse events [9].

Four-dimensional (4D) flow magnetic resonance imaging (MRI) is a relatively new radiological modality that provides comprehensive velocity and flow assessment in the examined area, which yielded excellent promising results in aortic and other cardiac pathologies [10, 11]. Although the application of 4D MRI in the porto-mesenteric vasculature could be hindered by the low-velocity flow, dual blood supply to the hepatic region, and the nearby respiratory movements, recent technological radiological advances have allowed detailed and precise examination in that difficult anatomical region [9, 12–15].

Therefore, 4D MRI can be used as a potential alternative to endoscopy in the risk stratification of upper GI varices [9, 16]. Although patients with portal hypertension and varices are encountered in hepatology and radiological clinics on a daily basis in Egypt, no previous Egyptian study has addressed the role of 4D MRI in such cases.

That was a fair motive for us to conduct the current investigation to assess the role of 4D MRI in the evaluation of upper GI varices and in the prediction of high-risk ones in patients with chronic liver disease.

## Methods

### Sample size calculation

We used the “IBM SPSS Sample Power” for sample size estimation. Motosugi et al reported that portal vein (PV) fractional flow change < 0 was 100% in high-risk patients vs 6% in non-risky cases (a difference of 94%) [9]. Assuming a 75% difference between risky and non-risky patients, a minimum sample of 38 patients needed to be included to achieve a 0.05 significance level and an 80% power.

### Study population

The current single-centre prospective cross-sectional study was conducted over a one-year period from January 2023 to January 2024. The research was designed for patients diagnosed with liver cirrhosis with or without hepatocellular carcinoma (HCC) and known to have oesophageal or gastric varices based on previous endoscopic examinations. Patient enrolment, procedures, and data collection did not start until we gained approval from our Institutional Review Board.

All patients were clinically assessed prior to the study procedures. That assessment focused on the aetiology and nature of their liver disease, previous endoscopic management of varices, and previous surgical procedures. Inclusion criteria include patients with chronic liver disease who were known to have either oesophageal or gastric varices, based on previous esophagogastroduodenoscopy. Exclusion criteria include patients aged less than 18 years, having prior endoscopic interventions for varices within the previous year, having general contraindications for MRI examination, patients with respiratory distress or abnormally high heart rate, or patients with PV thrombosis detected by previous ultrasound (US) or CT.

Thirty-eight patients known to have either oesophageal or gastric varices, based on previous esophagogastroduodenoscopy, were included in our study. All these cases underwent non-contrast conventional abdominal MRI, with 4D imaging of the porto-mesenteric vasculature, in addition to esophagogastroduodenoscopy. Patients were fasting for at least six hours before the MRI scan, as it was reported that 4D flow measurements of the porto-systemic circulation could vary between fasting and the postprandial states [17]. We performed the endoscopic assessment after the MRI, as the porto-mesenteric flow dynamics could change with endoscopic treatment (sclerotherapy or band ligation).

### 4D flow MRI protocol and post-processing

**Fig. 1:**
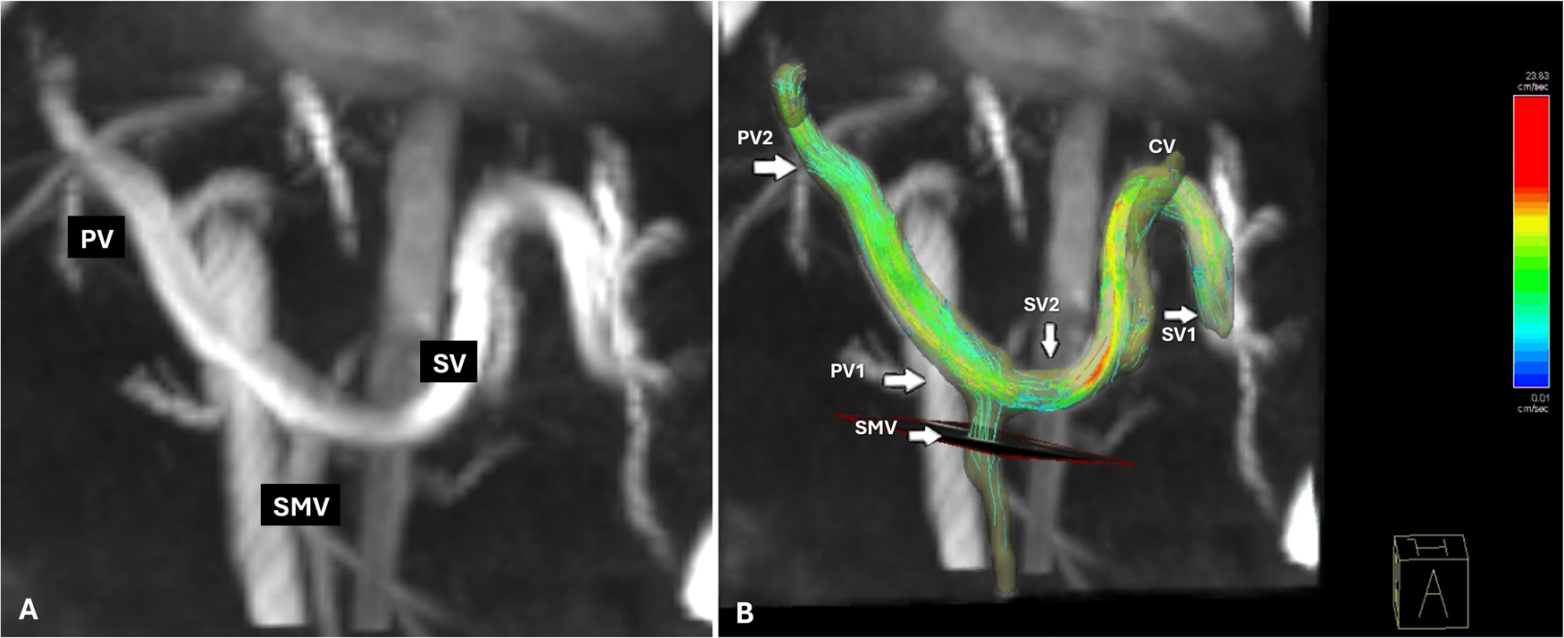
3D image (**A**) and 4D flow image (**B**) of the portal circulation show the anatomical landmarks where 4D flow parameters were measured. In this case coronary vein (CV) drains into the posterior aspect of the SV which is a normal anatomical variant. In this case PV1 average flow = 0.39 L/min, PV2 average flow = 0.2 L/min, SV1 average flow = 0.14 L/min, SV 2 average flow = 0.15 L/min, SMV average flow = 0.27 L/min, PV fractional average flow change = −0.5, SV fractional average flow change = −0.08. On endoscopy, the patient had grade III oesophageal varices which were classified as risky varices. PV, portal vein; SV, splenic vein; SMV, superior mesenteric vein; CV, coronary vein

MRI was done via a 1.5-T scanner (Siemens Magnetom Aera, Germany) using 8-channel phased array coils. The 4D flow was performed via phased contrast acquisition that covered the upper abdomen. The parameters used for obtaining the 4D flow images were as follows: field of view (FOV) = 380 mm, spatial resolution = 1.25 mm, slice thickness = 2.5 mm, time of repetition (TR)/time to echo (TE) = 42.7/2.68 ms, velocity encoding sensitivity = 20 cm/s, retrospective electrocardiogram (ECG) gating and adaptive respiratory gating were used. The 4D flow parameters were measured at five venous anatomical points as shown in Fig. 1 and corresponding Video S1. Two points were located on the PV: one at the PV-splenic vein confluence (PV1) and the other at the liver hilum (PV2). Two points were identified on the splenic vein (SV) confluence: one at the splenic hilum (SV1) and the other at the SV-superior mesenteric vein (SMV) confluence (SV2). The last point was specified for the SMV at the SV-SMV confluence.

The MRI images were assessed by two expert radiologists (reader 1 and reader 2), who are experienced in abdominal vascular imaging, in two separate sessions after randomization of cases. The radiologists were blinded to the diagnosis of the patients. The images were examined for the presence of varices, collaterals (with their location), liver size, spleen size, PV, SV, SMV, and coronary veins (CVs) diameter. The size of the liver and spleen were measured from the 4D flow source image (Time-averaged magnitude (MAG) image) in the coronal plane in maximum extension in the cranio-caudal direction. The 4D flow images were used to estimate three parameters at each of the previously mentioned five venous anatomical points, including peak flow (L/min), average flow (L/min), and peak velocity (cm/s). That provided us with 15 readings for the five anatomical points assessed. Moreover, the fractional change in blood flow across PV and SV was estimated twice, once using the average blood flow and the other using the peak blood flow via the equations published by Motosugi et al [9]. That was done to detect shunting from the PV to the CV and varices.

### Validation and consistency of the 4D flow MRI technique and measurements

The 4D flow of porto-mesentric circulation is challenging as it is susceptible to irregular heartbeats and abdominal respiratory movements. So, to make sure of the validity of our 4D flow MRI technique, seven patients from the 38 patients included in this study underwent Doppler US using a 3.5 MHz convex-array transducer (Toshiba Aplio 500, Toshiba Medical Systems, Tokyo, Japan), immediately after finishing the 4D flow MRI exam. The Doppler US was done by a third (reader 3) radiologist who was blinded to the results of the 4D flow. The flow was measured in Doppler US at the five anatomical points corresponding to those taken in the 4D flow MRI as shown in Fig. S1. Flow measurements from Doppler US were compared to those from the 4D flow MRI.

Interobserver reliability testing was done between the results of the two radiologists (reader 1 and reader 2) who separately interpreted the 4D flow MRI studies. The accuracy of 4D flow MRI measurements was also indirectly validated using conversion of mass principle by comparing the results of flow measurement of PV at the confluence (PV1) with the summation of flow measurements of the SMV and SV at the confluence (SMV + SV2) for all patients included in this study.

### Endoscopy

After the MRI assessment, the patients were referred to the Hepatology Department for a new endoscopic assessment. The endoscopist was blinded to the recent MRI findings. The location of the varices was recorded (oesophageal vs gastric). Patients with oesophageal varices were graded according to the Westaby classification (grade I: slightly protruded varices that were depressed by endoscopic insufflation; grade II: varices < 50% of the oesophageal lumen, or grade III: varices occupying > 50% of the oesophageal lumen with a confluent appearance [18]. The patient was considered to have risky varices if the endoscopist found red wale marks, cherry red spots, diffuse redness, hematocystic spots, large varices, or evidence of recent bleeding [19–22].

### Statistical analysis

The SPSS software was used to analyse the previously collected data. The two-group comparison (based on variceal location and risk stratification) was done using the following tests: sample *t*-test (for means), Mann–Whitney test (for medians), and Chi-square test (Fischer exact or Monte Carlo tests) for frequencies. According to the oesophageal variceal grade, three groups were obtained. The categorical variables (frequencies) were compared using the same previous tests, while one-way ANOVA and Kruskal–Wallis tests were used for means and medians, respectively. Receiver operating characteristic (ROC) curves were also obtained, and regression analysis was performed to reveal predictors of risky varices. Correlation was also done between the oesophageal variceal grade and the MRI parameters. Any obtained *p* value < 0.05 was considered statistically significant. Interobserver agreement and validation of the 4D flow measurements were done using intraclass correlation coefficients (ICCs) at 95% confidence intervals (CIs) [23].

## Results

The mean age of the included patients was 57.63 years, with a higher predominance for men over women (68.4% vs 31.6%, respectively). HCV was the most common cause of liver disease (63.2%). Other aetiologies included bilharziasis, and autoimmune liver disease. Four cases out of the 38 (10.5%) had HCC. Via endoscopy, oesophageal varices were detected in 30 cases (78.9%), whereas the remaining cases had gastric varices. Risky varices were encountered in 12 cases (31.6%). In patients with oesophageal varices, grade I, II, and III were detected in 66.7%, 20%, and 13.3% of cases, respectively. Liver and spleen sizes had mean values of 16.69 cm and 13.09 cm, respectively. The mean diameter of the main PV was 10.85 mm. Regarding collaterals detected on MRI examination, most cases (42.11%) had no collaterals. The splenic hilar collaterals were the most collaterals among our patients (26.32%). Patients’ demographics, and endoscopic and MRI data were summarized in supplementary Table S1.

As shown in Table 1, on comparison between patients with oesophageal vs gastric varices, we noted a significant increase in splenic size, as well as an increase in the prevalence of collaterals in patients with gastric varices (*p* = 0.037 and < 0.001, respectively). Most of the measured flow, velocity, and fractional change parameters did not show notable statistical differences between the two groups, apart from four measurements, which increased significantly in patients with oesophageal compared to gastric varices. These significant parameters included SV1 average flow (0.4 vs 0.14 L/min, respectively, *p* = 0.022), SV2 average flow (0.29 vs 0.08 L/min, respectively, *p* < 0.001), SV2 peak flow (0.41 vs 0.11 L/min, respectively, *p* < 0.001), and SV2 peak velocity (14.77 vs 8.55 cm/s, respectively, *p* = 0.001).

**Table 1:** Comparison between patients with oesophageal vs gastric varices

Variables	Oesophageal, (*N* = 30)	Gastric, (*N* = 8)	Test of significance	*p* value
Spleen size	16.08 ± 2.56	19.33 ± 5.66	*t* = −2.187	0.037*
Liver size	13.49 ± 3.01	11.60 ± 2.94	*t* = 1.582	0.122
PV diameter	10.71 ± 1.94	11.38 ± 3.65	*t* = −0.709	0.483
SV diameter	7.5 ± 2.1	8.7 ± 2.3	*t* = 1.207	0.237
SMV diameter	7.5 ± 1.8	8 ± 2.4	*t* = 0.847	0.404
CV diameter	4.4 ± 2.2	4.3 ± 0.27	*t* = −0.710	0.483
Main collateral on MRI				
No collaterals	14 (75%)	2 (25%)	MC = 21.454	< 0.001*
Epigastric	0 (0%)	2 (25%)		
Oesophageal	4 (13.3%)	0 (0%)		
Fundic	0 (0%)	2 (25%)		
Peri-splenic	2 (6.7%)	2 (25%)		
Splenic hilar	10 (33.3%)	0 (0%)		
4D parameters at the assessed five anatomical points				
PV1 average flow (L/min)	0.66 (0.3–1.44)	0.47 (0.42–0.77)	*z* = −1.578	0.115
PV1 peak flow *(*L/min*)*	0.78 (0.4–1.68)	0.58 (0.48–1.18)	*z* = −1.721	0.085
PV1 peak velocity (cm/s)	15.7 (11.5–24.26)	11.4 (11.1–24)	*z* = − 0.547	0.566
PV2 average flow (L/min)	0.42 (0.14–1.07)	0.32 (0.19–0.59)	*z* = −1.293	0.196
PV2 peak flow (L/min)	0.48 (0.2–1.22)	0.36 (0.27–0.83)	*z* = −1.004	0.315
PV2 peak velocity (cm/s)	13.6 (8.6–22.6)	13.9 (11.6–26.4)	*z* = −0.431	0.666
SMV average flow (L/min)	0.23 (0.02–0.48)	0.26 (0.18–0.31)	*z* = −0.861	0.389
SMV peak flow (L/min)	0.29 (0.07–0.57)	0.35 (0.19–0.51)	*z* = −0.646	0.518
SMV peak velocity (cm/s)	9.40 (2.5–18.9)	9.20 (7–41.8)	*z* = −0.431	0.666
SV1 average flow (L/min)	0.40 (0–0.85)	0.14 (0.07–0.36)	*z* = −2.297	0.022*
SV1 peak flow (L/min)	0.53 (0–0.93)	0.23 (0.09–0.37)	*z* = −1.723	0.085
SV1 peak velocity (cm/s)	14.59 (0–21.5)	10.75 (6.4–38.8)	*z* = −0.718	0.473
SV2 average flow (L/min)	0.29 (0.12–1.04)	0.08 (0.04–0.24)	*z* = −3.659	< 0.001*
SV2 peak flow (L/min)	0.41 (0.13–1.27)	0.11 (0.08–0.28)	*z* = −3.729	< 0.001*
SV2 peak velocity (cm/s)	14.77 (6–33)	8.55 (7.5–11)	*z* = −3.444	0.001*
PV fractional average flow change	0.02 (−0.78:0.64)	−0.08 (−0.54:0.66)	*z* = −0.143	0.866
PV fractional peak flow change	−0.03 (−0.68:0.38)	−0.23 (−0.41:0.41)	*z* = −0.143	0.866
SV fractional average flow change	−0.13 (−2.07:1)	−0.61 (−1.36:0)	*z* = −1.759	0.073
SV fractional peak flow change	−0.07 (−1.04:1)	−0.25 (−2.33:0.11)	*z* = −1.865	0.062

*t* Independent samples *t*-test, *z* Mann–Whitney *U*-test, MC Montecarlo test

*PV* portal vein, *SV* splenic vein, *SMV* superior mesenteric vein

* Statistically significant (*p* < 0.05)

Patients with grades II and III had a higher prevalence of collaterals compared to grade I patients (*p* = 0.003). Patients with higher variceal grades had significantly lower PV2 average flow (*p* = 0.018), PV2 peak flow (*p* = 0.049), PV fractional average flow change (*p* = 0.001), and PV fractional peak flow change (*p* < 0.001). In contrast, the same patients expressed significantly higher PV1 average flow (*p* = 0.022), SMV peak flow (*p* = 0.038), and SV2 peak velocity (*p* = 0.047) compared to grade I cases. Table 2 illustrates the previous data.

**Table 2:** Comparison between the three grades of oesophageal varices

Variables	Grade 1, (*N* = 20)	Grade 2, (*N* = 6)	Grade 3, (*N* = 4)	Test of significance	*p* value
Spleen size	16.38 ± 2.62	15.33 ± 2.84	16 ± 2.31	*F* = 0.344	0.712
Liver size	12.60 ± 3.21	15.77 ± 1.19	14.50 ± 1.73	*F* = 3.251	0.054
PV diameter	10.16 ± 2.12	11.33 ± 0.52	12.50 ± 0.58	*F* = 3.251	0.054
The main collateral on MR					
No collaterals	14 (70%)	0 (0%)	0 (0%)	MC = 19.900	0.003*
Epigastric	0 (0%)	0 (0%)	0 (0%)		
Oesophageal	0 (0%)	2 (33.3%)	2 (50%)		
Fundic	0 (0%)	0 (0%)	0 (0%)		
Peri-splenic	2 (10%)	0 (0%)	0 (0%)		
Splenic hilar	4 (20%)	4 (66.7%)	2 (50%)		
4D parameters at the assessed five anatomical points					
PV1 average flow (L/min)	0.62 (0.3–0.71)	0.76 (0.67–1.44)	0.80 (0.39–1.20)	kw = 7.598	0.022*
PV1 peak flow (L/min)	0.77 (0.4–0.86)	0.86 (0.75–1.68)	0.95 (0.55–1.36)	kw = 4.496	0.106
PV1 peak velocity (mL/s)	14 (11.5–24.26)	15.7 (14.9–18.9)	19.25 (17.4–21.1)	kw = 4.896	0.086
PV2 average flow (L/min)	0.55 (0.14–1.07)	0.35 (0.33–0.39)	0.31 (0.21–0.41)	kw = 8.043	0.018*
PV2 peak flow (L/min)	0.68 (0.2–1.22)	0.46 (0.38–0.59)	0.35 (0.26–0.44)	kw = 6.018	0.049*
PV2 peak velocity (cm/s)	14 (8.6–22.6)	12.6 (10–16.30)	15.55 (12.8–18.30)	kw = 2.177	0.337
SMV average flow (L/min)	0.14 (0.02–0.48)	0.23 (0.20–0.45)	0.29 (0.27–0.31)	kw = 4.910	0.086
SMV peak flow (L/min)	0.23 (0.07–0.54)	0.29 (0.24–0.51)	0.45 (0.34–0.57)	kw = 6.521	0.038*
SMV peak velocity (cm/s)	9.40 (2.5–16)	8.4 (7.9–12.6)	14.1 (9.3–18.9)	kw = 2.176	0.337
SV1 average flow (L/min)	0.35 (0–0.57)	0.49 (0.23–0.55)	0.49 (0.14–0.85)	kw = 0.780	0.677
SV1 peak flow (L/min)	0.44 (0–0.65)	0.57 (0.26–0.72)	0.57 (0.22–0.93)	kw = 1.351	0.509
SV1 peak velocity (cm/s)	14.59 (0–18.2)	17.9 (11.1–21.5)	16.65 (12.9–20.4)	kw = 4.554	0.103
SV2 average flow (L/min)	0.29 (0.12–0.48)	0.32 (0.29–1.04)	0.21 (0.15–0.28)	kw = 5.492	0.064
SV2 peak flow (L/min)	0.40 (0.13–0.54)	0.41 (0.34–1.27)	0.34 (0.23–0.46)	kw = 2.117	0.347
SV 2 peak velocity (cm/s)	14.3 (6–33)	15.6 (15.1–20.4)	18.79 (14.77–22.8)	kw = 6.126	0.047*
PV Fractional average flow change	0.23 (−0.70: 0.64)	−0.32 (−0.78: −0.25)	−0.39 (−0.50: −0.29)	kw = 13.741	0.001*
PV fractional peak flow change	0.09 (−0.64: 0.38)	−0.40 (−0.67: −0.29)	−0.56 (−0.68: −0.45)	kw = 15.484	< 0.001*
SV fractional average flow change	−0.13 (−0.84: 1)	0.21 (−0.54: 0.47)	−0.99 (−2.07: 0.08)	kw = 1.708	0.426
SV fractional peak flow change	− 0.07 (−0.33: 1)	0.25 (−0.4: 0.43)	−0.50 (−1.04: 0.03)	kw = 1.653	0.438

*F* One-Way ANOVA test, MC Montecarlo test, and KW Kruskal–Wallis test

*PV* portal vein, *SV* splenic vein, *SMV* superior mesenteric vein

* Statistically significant (*p* < 0.05)

**Fig. 2:**
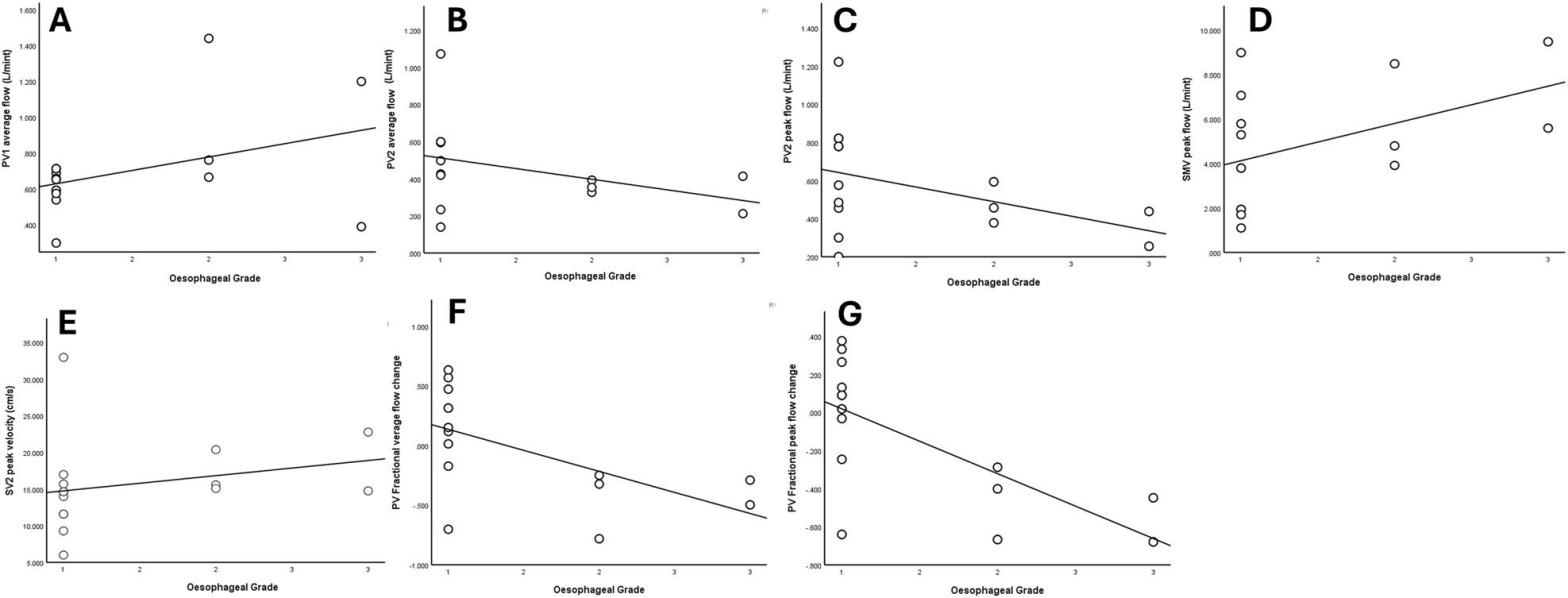
Correlation between oesophageal grade and **A** PV1 average flow, **B** PV2 average flow, **C** PV2 peak flow, **D** SMV peak flow, **E** SV2 peak velocity, **F** PV fractional average flow, and **G** PV fractional peak flow

Oesophageal variceal grade had a significant positive correlation with PV1 average flow (*r* = 0.362, *p* = 0.049), SMV peak flow (*r* = 0.459, *p* = 0.011), and SV2 peak velocity (*r* = 0.454, *p* = 0.012), whereas it had a significant negative correlation with PV2 average flow (*r* = −0.522, *p* = 0.003), PV2 peak flow (*r* = 0.431, *p* = 0.017), PV fractional average flow change (*r* = −0.67, *p* < 0.001), and PV fractional peak flow change (*r* = −0.73, *p* < 0.001) (not expressed in the tables) (Fig. 2 and supplementary Table S2).

As expressed in Table 3, patients with risky varices had larger liver sizes (*p* = 0.009) and a higher prevalence of venous collaterals (*p* = 0.002). Patients with risky varices had higher SV2 peak velocity compared to non-risky cases. On the other hand, the high-risk cases expressed significantly lower PV2 average flow, PV2 peak flow, PV fractional average flow change, and PV fractional peak flow change compared to non-risky ones. There was no significant difference between patients with risky varices and those with non-risky ones regarding PV, SV, SMV, or CV diameter.

**Table 3:** Comparison between risky and non-risky varices

Variables	No risk, [*N* = 26]	Risky, [*N* = 12]	Test of significance	*p* value
Spleen size	17.18 ± 3.79	15.60 ± 2.53	*t* = 1.200	0.240
Liver size	12.23 ± 3.22	14.95 ± 1.53	*t* = −2.768	0.009*
PV diameter	10.70 ± 2.63	11.17 ± 1.64	*t* = −0.563	0.577
SV diameter	8.2 ± 2.5	7.5 ± 1.5	*t* = 1.77	0.087
SMV diameter	7.2 ± 2	8.2 ± 1.4	*t* = −1.89	0.070
CV diameter	4 ± 1.9	4.9 ± 2.1	*t* = −0.78	0.439
The main collateral on MR				
No collaterals	14 (53.8%)	2 (25%)	MC = 18.793	0.002*
Epigastric	2 (7.7%)	0 (0%)		
Oesophageal	0 (0%)	4 (33.3%)		
Fundic	2 (7.7%)	0 (0%)		
Splenic	4 (15.4%)	0 (0%)		
Splenic hilar	4 (15.4%)	6 (50%)		
4D parameters at the assessed five anatomical points				
PV1 average flow (L/min)	0.59 (0.3–0.77)	0.71 (0.39–1.44)	*z* = −1.258	0.208
PV1 peak flow *(*L/min*)*	0.76 (0.4–1.18)	0.80 (0.48–1.68)	*z* = −1.006	0.314
PV1 peak velocity (mL/s)	14 (11.1–24.26)	16.7 (14.9–21.1)	*z* = −1.888	0.059
PV2 average flow (L/min)	0.50 (0.14–1.07)	0.34 (0.19–0.41)	*z* = −3.023	0.003*
PV2 peak flow (L/min)	0.58 (0.2–1.22)	0.41 (0.26–0.59)	*z* = −2.265	0.023*
PV2 peak velocity (cm/s)	13.8 (8.6–26.4)	13.4 (10–18.3)	*z* = −0.765	0.450
SMV average flow (L/min)	0.22 (0.02–0.48)	0.25 (0.18–0.45)	*z* = −1.384	0.166
SMV peak flow (L/min)	0.28 (0.07–0.54)	0.31 (0.19–0.57)	*z* = −1.322	0.186
SMV peak velocity (cm/s)	9.40 (2.5–41.8)	8.85 (7–18.9)	*z* = −0.126	0.900
SV1 average flow (L/min)	0.24 (0–0.57)	0.43 (0.14–0.85)	*z* = −1.637	0.102
SV1 peak flow (L/min)	0.35 (0–0.65)	0.47 (0.22–0.93)	*z* = −1.700	0.089
SV1 peak velocit*y* (cm/s)	14.59 (0–38.8)	15.4 (11.1–21.5)	z = −1.763	0.078
SV2 average flow (L/min)	0.24 (0.04–0.48)	0.25 (0.15–1.04)	*z* = −1.447	0.148
SV2 peak flow (L/min)	0.29 (0.08–0.54)	0.37 (0.23–1.27)	*z* = −1.132	0.258
SV2 peak velocity (cm/s)	11.6 (6–33)	15.35 (11–22.8)	*z* = −2.517	0.012*
PV fractional average flow change	0.16 (−0.70: 0.66)	−0.41 (−0.78: −0.25)	*z* = −4.026	< 0.001*
PV fractional peak flow change	0.09 (−0.64: 0.41)	−0.43 (−0.68: −0.29)	*z* = −4.277	< 0.001*
SV fractional average flow change	−0.14 (−1.36: 1)	−0.21 (−2.07: 0.47)	*z* = −0.252	0.801
SV fractional peak flow change	−0.07 (−2.33: 1)	−0.16 (−1.04: 0.43)	*z* = −0.629	0.529

The varices were categorized as risky and non-risky ones depending on endoscopic findings

*t* Independent samples *t*-test, FET Fischer’s exact test, *z* Mann–Whitney *U*-test, MC Montecarlo test

*PV* portal vein, *SV* splenic vein, *SMV* superior mesenteric vein

* Statistically significant (*p* < 0.05)

**Fig. 3:**
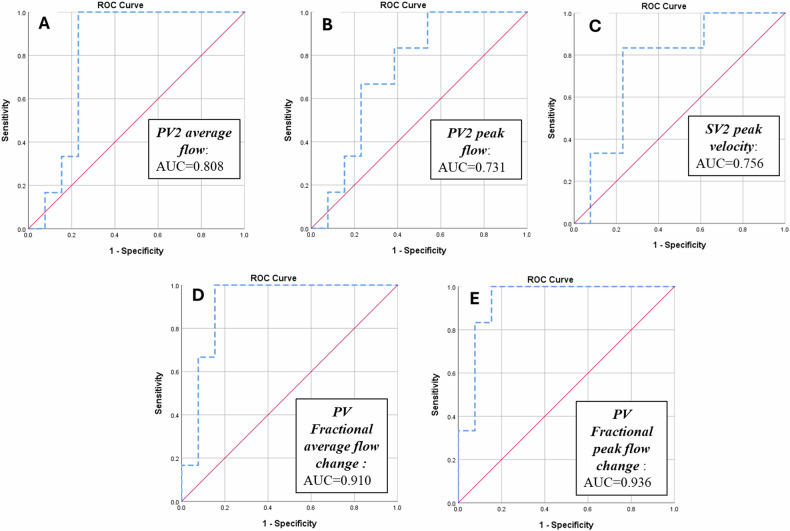
ROC analysis for PV2 average flow (**A**), PV2 peak flow (**B**), SV2 peak velocity (**C**), PV Fractional average change (**D**), and PV Fractional peak flow change (**E**) in the prediction of risk varices

ROC curve analysis revealed the following findings for the prediction of risky varices (Fig. 3):

- PV2 average flow had sensitivity and specificity of 100% and 76.9%, respectively, when a cut-off value of < 0.417 L/min was applied (accuracy = 84.6%, area under the curve (AUC) = 0.808, *p* = 0.003).
- PV2 peak flow had an 83.3% sensitivity and 61.4% specificity, with an accuracy of 78.6% (cut-off value < 0.471 L/min, AUC = 0.731, *p* = 0.024).
- Using a cut-off value of > 14.71 cm/s, SV2 velocity had a sensitivity and specificity of 83.3% and 76.9%, respectively, with an accuracy of 81.2% (AUC = 0.756, *p* = 0.012).
- PV fractional average flow change has a 100% sensitivity and an 84.6% specificity when a cut-off value of < − 0.210 was applied (accuracy = 89.2%, AUC = 0.910, *p* < 0.001).
- PV fractional peak flow change had an 83.3% sensitivity and 92.3% specificity when we used a cut-off value of < − 0.348 (accuracy = 87.1%, AUC = 0.936, *p* < 0.001).

These data are expressed in the Supplementary Table S3.

Univariate regression analysis revealed that enlarged liver size, lower P2 average flow, lower P2 peak flow, lower PV fractional average flow change, and lower PV fractional peak flow change were significant risk factors for high-risk varices. Nonetheless, only the last two parameters maintained their significance in the multivariate analysis (Table 4).

**Table 4:** Regression analysis for prediction of risky varices

	Univariate regression analysis				Multivariate regression analysis			
	*p* value	Odds ratio	95% CI for odds ratio		95% CI for odds ratio	Odds ratio	95% CI for odds ratio	
			Lower	Lower			Lower	Upper
Liver size	0.025*	1.595	1.059	2.401	0.766	1.174	0.622	1.572
PV2 average flow (L/min)	0.021*	0.002	0.000	0.390	0.078	0.138	0.016	1.193
PV2 peak (L/min)	0.028*	0.012	0.000	0.625	0.603	0.762	0.273	2.125
SV2 peak velocity (cm/s)	0.136	1.094	0.972	1.230				
PV fractional average flow change	0.003*	0.004	0.000	0.152	0.005*	0.483	0.230	0.846
PV fractional peak flow change	0.003*	0.000	0.000	0.072	0.001*	0.546	0.238	0.762

The varices were categorized as risky ones depending on endoscopic findings

*CI* confidence interval, *OR* odd’s ratio, *PV* portal vein, *SV* splenic vein, *SMV* superior mesenteric vein

* Statistically significant (*p* < 0.05)

Validation of the 4D flow measurements was done in this study by comparing the flow results at the five anatomical points derived from the 4D flow MRI with those derived from Doppler US in 7 patients among the 38 ones enroled in this study. Regarding agreement between the 4D flow MRI results and Doppler US results, we found good reliability for the PV1 average flow (ICC = 0.843, 95% CI = 0.085–0.973, *p* value = 0.020), good reliability for PV2 average flow (ICC = 0.870, 95% CI = 0.423–0.976, *p* value = 0.002), excellent reliability for SV1 average flow (ICC = 0.974, 95% CI = 0.850–0.996, *p* value < 0.001), moderate reliability for SV2 average flow (ICC = 0.552, 95% CI = 0.160–0.923, *p* value = 0.17) and good reliability for SMV average flow (ICC = 0.890, 95% CI = 0.493–0.980, *p* value = 0.002). In addition, we validated the 4D flow measurements indirectly depending on the concept of continuity (conversion of mass). Comparison of the average flow in PV2 and the summation of the average flow in SV2 and SMV revealed good reliability (ICC = 0.842, 95% CI = 0.564–0.943, *p* value < 0.001).

Interobserver agreement between the two radiologist (reader 1 and reader 2) who interpreted the 4D flow MRI revealed good reliability for the PV1 average flow (ICC = 0.771, 95% CI = 0.350–0.933, *p* value = 0.002), excellent reliability for PV1 peak velocity (ICC = 0.947, 95% CI = 0.802–0.986, *p* value < 0.001), good reliability for PV2 average flow (ICC = 0.873, 95% CI = 0.360–0.934, *p* value = 0.002), excellent reliability for PV2 peak velocity (ICC = 0.983, 95% CI = 0.938–0.995, *p* value < 0.001), good reliability for SV1 average flow (ICC = 0.797, 95% CI = 0.246–0.945, *p* value = 0.009), good reliability for SV1 peak velocity (ICC = 0.811, 95 % CI = 0.530–0.921, *p* value < 0.001), moderate reliability for SV2 average flow (ICC = 0.679, 95% CI = 0.169–0.902, *p* value = 0.008), good reliability for SV2 peak velocity (ICC = 0.884, 95% CI = 0.626–0.967, *p* value < 0.001), good reliability for SMV average flow (ICC = 0.781, 95% CI = 0.373–0.936, *p* value = 0.001) and excellent reliability for SMV velocity (ICC = 0.989, 95% CI = 0.959–0.957, *p* value < 0.001).

## Discussion

Our study assessed the role of 4D MRI in risk stratification of patients with cirrhosis-related upper GI varices. That poses a great advantage in favour of our research, especially since the international literature is also poor with studies handling the same perspective.

Giannini et al reported the poor accuracy of serum markers used to predict high-risk varices, despite their simplicity compared to other methods [24]. Other researchers used hepatic and splenic elastography for the same purpose. Nonetheless, not all patients with stiff livers develop risky varices [25–28]. Furthermore, some studies documented the application of the US in the assessment of portal blood flow and the prediction of varices. However, limited FOV, complex anatomy, and operator dependency are the major drawbacks of US when used for that purpose [29, 30].

Endoscopy is a minimally invasive technique that is routinely used in such cases. However, it may lead to some complications. The measurement of “hepatic venous wedge pressure” provides a direct measurement of the venous pressure. However, it is an invasive and expensive procedure that cannot be routinely used in clinical practice [16]. Although 4D MRI cannot provide direct pressure measurements, it can provide accurate information about variceal morphology along with venous blood direction, rate, and velocity using a single non-invasive examination [16, 19, 31, 32].

To our knowledge, this is the first study to evaluate the efficacy of the 4D flow in differentiating between gastric and oesophageal varices depending on the 4D flow parameters and also to assess the difference of the 4D flow parameters among different grades of oesophageal varices. Most previous studies focused on differentiating between high-risk and non-risky varices [9, 19]. Furthermore, we correlated those parameters with the oesophageal varices grading.

Four measurements increased significantly in patients with oesophageal compared to gastric varices. These significant parameters included SV1 average flow (*p* = 0.022), SV2 average flow (*p* < 0.001), SV2 peak flow (*p* < 0.001), and SV2 peak velocity (*p* = 0.001). The significant decline of SV2 average and peak flows in patients with gastric varices compared to the oesophageal ones has a reasonable explanation. Gastric varices are the consequence of collateral formation between splenic and gastric veins [33]. Therefore, the blood that emerged from the spleen through the SV must have been diverted through these collaterals, leading to these changes compared to oesophageal varices patients, which depend mainly on the CV and its related collaterals.

In our study, the reader could see that PV1 (reflecting portal inflow) was higher in grade-III patients. On the other hand, PV2 average and peak flows (measured at liver hilum), along with portal fractional flow changes, decreased significantly in the same patient group. That means that a larger portion of blood flow was diverted after the inflow through the collaterals, leading to more dilatation of the varices detected during endoscopy. That was also confirmed by subsequent statistical correlation.

Our findings revealed that patients with risky varices had significantly lower PV2 average flow, PV2 peak flow, PV fractional average flow change, and PV fractional peak flow change compared to non-risky cases. The reader could notice that both fractional flow changes had the highest AUC when performing ROC analysis (AUC = 0.91 and 0.936, respectively), and they were also significant predictors in the regression analysis. As the fractional flow changes assess blood shunting through the CV [9], it is expected to find these parameters more sensitive than others when assessing risky varices, as 80% of varices are supplied by that vein [34, 35]. In agreement with our findings, Motosugi et al used 4D MRI to predict high-risk varices in their study, which included 23 participants. The AUC analysis revealed a moderate association between high-risk varices and either PV2 average flow (AUC = 0.723, CI = 0.326–0.934) or fractional portal flow change (AUC = 0.955, CI = 0.72–0.994). Additionally, PV2 < 0.7 L/min was a significant predictor for high-risk varices on the univariate analysis (*p* = 0.008). PV fractional flow change < 0 maintained its significance on both univariate and multivariate analyses (*p* < 0.001), and it had a 100% sensitivity and 94% specificity to detect high-risk variceal patients [9].

Ji et al agreed with our findings regarding the insignificant impact of portal blood velocity on the prediction of risky varices, and that was evident in their average and peak velocities (*p* = 0.282 and 0.112, respectively). However, the same authors found a significant rise in portal flow volume in association with high-risk varices (14.31 ± 3.64 vs 10.77 ± 3.37 mL/cycle in no or low-risk varices, *p* < 0.001). They attributed their findings to the increased splanchnic blood flow and systemic vasodilation in chronic liver disease, which in turn increased portal venous inflow [19]. Additionally, Burkart et al found that increased portal venous flow was independently associated with variceal haemorrhage in both univariate and multivariate analyses (*p* = 0.001 and 0.006, respectively) when they used 2D MRI for the same purpose [36]. We had a reasonable explanation for that conflict. The previous two studies must have assessed the PV flow at its origin (behind the pancreatic neck), which is expected to increase due to vasodilation and increased splanchnic blood flow. Surprisingly, our PV1 showed the same findings, although it did not reach statistical significance. However, we noticed a decrease in blood flow when assessing it at the liver hilum. That means that the majority of the portal inflow has already been diverted through the venous collaterals before reaching the liver hilum, which indicates increased blood flow through the collaterals, which are responsible for bleeding. We think that hilar flow measurement and its comparison to the inflow measurement would be more accurate than the inflow itself, as the former more accurately reflects the amount of venous blood diverted through collaterals to the varices, making it more susceptible to bleeding by the increased flow inside.

Previous studies that used computed tomography (CT) to predict the risk of oesophageal variceal haemorrhage reported that patients with larger diameter of CV diameter are at higher risk of oesophageal variceal haemorrhage [37]. Previous 4D flow MRI studies didn’t evaluate the accuracy of CV diameter in predicting risky oesophageal varices [9]. In our study, we found no significant difference between patients with risky varices and those with non-risky ones regarding the diameter of CV. This finding makes the morphologic assessment of the 4D flow questionable and needs further research to confirm its feasibility.

In our study, we found a significantly larger liver size in patients with risky varices compared to those without risky varices. This can be explained by the fact that four of the patients with risky varices had HCC and had an enlarged liver size of (17 ± 3 cm).

Our study has some limitations. First, the small sample size is the main limitation of our research. Second, we should have assessed the impact of therapeutic intervention or medical treatment on porto-mesenteric hemodynamic parameters. Further studies should address the previous drawbacks. Third, the application of 4D flow MRI is challenging, including the need for specialized equipment, software, and high patient compliance; these limitations can be critical in the technique’s widespread use and accessibility in clinical practice.

## Conclusion

Based on certain porto-mesenteric blood flow, velocity, and fractional flow change parameters, 4D flow MRI can have excellent sensitivity and high specificity for the prediction of high-risk variceal patients. It also can give an excellent idea about the type of varices either oesophageal or gastric and also the grade of oesophageal varices. That makes 4D flow MRI a potential alternative for minimally invasive endoscopy to predict patients with high-risk varices.

## Supplementary information

ELECTRONIC SUPPLEMENTARY MATERIAL

Video S1

## Data Availability

All data generated or analysed during this study are included in this published article.

## Abbreviations

4D: Four-dimensional
AUC: Area under the curve
CT: Computed tomography
CV: Coronary vein
ECG: Electrocardiogram
FOV: Field of view
GI: Gastrointestinal
HCC: Hepatocellular carcinoma
HCV: Hepatitis C virus
MRI: Magnetic resonance imaging
PV: Portal vein
ROC: Receiver operating characteristic
SMV: Superior mesenteric vein
SV: Splenic vein
TR/TE: Time of repetition/time to echo
US: Ultrasound
