# Supplementary material for: Porto-mesenteric four-dimensional flow MRI: a novel non-invasive technique for assessment of gastro-oesophageal varices
Source: Insights Imaging. 2024 Sep 27;15:231. doi: 10.1186/s13244-024-01805-6 (PMC11436693; doi:10.1186/s13244-024-01805-6)
Supplement: Supplementary file 1 — ELECTRONIC SUPPLEMENTARY MATERIAL [file 13244_2024_1805_MOESM1_ESM.pdf]

Porto-mesenteric Four-Dimensional Flow MRI: a novel non-invasive technique for assessment of gastro-oesophageal varices.

ELECTRONIC SUPPLEMENTARY MATERIAL

Supplementary Table (S1): Demographic, endoscopic, and conventional radiological data in the study cases.

**Variables**	**Study cases (N = 38)**
**Age (years)**	57.63 ± 14.40
**Gender**	
Male	26 (68.4%)
Female	12 (31.6%)
**Causes of liver diseases**	
Autoimmune hepatitis	6 (15.8%)
Bilharziasis	8 (21.1%)
HCV	24 (63.2%)
**Presence of HCC**	
Yes	4 (10.5%)
No	34 (89.5%)
**Endoscopic findings**	
**Type of varices**	
Esophageal	30 (78.9%)
Gastric	8 (21.1%)
**Grades of esophageal varices (n= 30)**	
Grade I	20 (66.7%)
Grade II	6 (20%)
Grade III	4 (13.3%)
**Risk stratification**	
No risk	26 (68.4%)

High risk	12 (31.6%)
**Radiological data**	
Spleen size (cm)	16.69 ± 3.48
Liver size (cm)	13.09 ± 3.06
Portal vein diameter (mm)	10.85 ± 2.35
**Site of collaterals**	
No collaterals	16 (42.11%)
Epigastric	2 (5.26%)
Esophageal	4 (10.53%)
Fundic	2 (5.26%)
Peri-splenic	4 (10.53%)
Splenic hilar	10 (26.32%)

Continuous data expressed as mean ± SD and median (range)

Categorical data expressed as Number (%)

Supplementary table (S2): Correlation between oesophageal grading and other variables in the study.

		Esophageal Grade
PV1 average flow	rs	0.362
	p	0.049*
PV2 average flow	rs	-0.522
	p	0.003*
PV2 peak flow	rs	-0.431
	p	0.017*
SMV peak flow	rs	0.459
	p	0.011*
SV2 peak velocity	rs	0.454
	p	0.012*
PV Fractional average flow change	rs	-0.670
	p	< 0.001*
PV Fractional peak flow change	rs	-0.730**
	p	< 0.001*

Test of significance is Spearman's correlation.

*: significant p value.

Supplementary table (S3): Diagnostic values of significant parameters to detect high-risk variceal patients.

Diagnostic criteria	PV2 average flow (L/min)	PV2 peak flow (L/min)	SV2 peak velocity (cm/sec)	PV Fractional average flow change	PV Fractional peak flow change
**AUC**	0.808	0.731	0.756	0.910	0.936
**Cut off point**	< 0.417	< 0.471	> 14.71	< - 0.210	< - 0.348
**Sensitivity**	100 %	83.3 %	83.3 %	100 %	83.3 %
**Specificity**	76.9 %	61.4 %	76.9 %	84.6 %	92.3 %
**NPV**	80.3 %	66.3 %	72.4 %	86.3 %	90.4 %
**PPV**	100 %	80.4 %	78.7 %	100 %	84.1 %
**Accuracy**	84.6 %	78.6 %	81.2 %	89.2 %	87.1 %
**P**	**0.003***	**0.024***	**0.012***	**< 0.001***	**< 0.001***

AUC: area under the curve, NPV: Negative predictive value, PPV: Positive predictive value

*: significant p value (< 0.05).

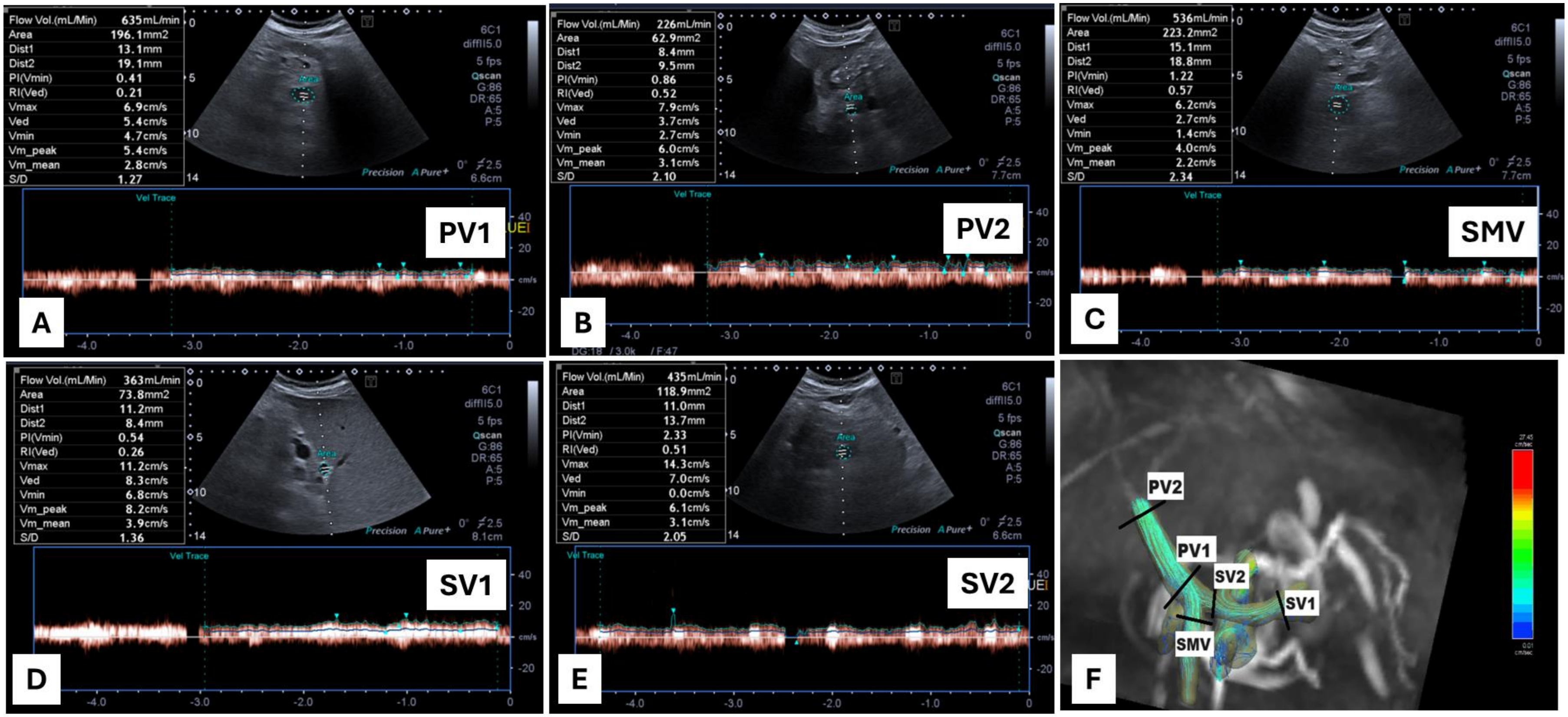

Figure (S1): Doppler Us at the level of PV1 (A), PV2 (B), SMV (C), SV1 (D) and SV2 (E) and 4D flow image (F) of the portal circulation showing the anatomical landmarks where 4D flow parameters were measures corresponding to the levels taken in doppler US .In this case PV1 was 0.623L/mint in doppler and 0.682L/mint and in 4D flow, PV2= 0.226L/mint in doppler and 0.21 L/mint in 4D flow, SMV=0.536L/mint in doppler and 0.441 L/mint, SV1= 0.363L/mint in doppler and 0.217L/mint on 4D flow , SV2= 0.435L/mint in doppler and 0.329L/mint in 4D flow. So according to Doppler and 4D flow the PV fractional flow change= -0.77 and 0.72 respectively. On endoscopy the patient had grade III oesophageal varices which was classified as risky varices. PV= portal vein, SV= splenic vein, SMV= superior mesenteric vein.
